# 2BC Non-Structural Protein of Enterovirus A71 Interacts with SNARE Proteins to Trigger Autolysosome Formation

**DOI:** 10.3390/v9070169

**Published:** 2017-07-04

**Authors:** Jeffrey K. F. Lai, I-Ching Sam, Pauline Verlhac, Joël Baguet, Eeva-Liisa Eskelinen, Mathias Faure, Yoke Fun Chan

**Affiliations:** 1 Department of Medical Microbiology, Faculty of Medicine, University Malaya, 50603 Kuala Lumpur, Malaysia; jefferslai@gmail.com (J.K.F.L.); jicsam@ummc.edu.my (I.-C.S.); 2 CIRI, International Center for Infectiology Research, Université de Lyon, 69007 Lyon, France; pauline.verlhac@inserm.fr (P.V.); joel.baguet@inserm.fr (J.B.); mathias.faure@inserm.fr (M.F.); 3 INSERM, U1111, 69007 Lyon, France; 4 CNRS, UMR5308, 69007 Lyon, France; 5 Ecole Normale Supérieure de Lyon, 69007 Lyon, France; 6 Université Lyon 1, Centre International de Recherche en Infectiologie, 69365 Lyon, France; 7 Department of Biosciences, Division of Biochemistry and Biotechnology, University of Helsinki, 00014 Helsinki, Finland; eeva-liisa.eskelinen@helsinki.fi; 8 Institut Universitaire de France, 75231 Paris, France; 9 Equipe labellisée Fondation pour la Recherche Médicale FRM, 75007 Paris, France

**Keywords:** picornavirus, enterovirus, enterovirus A71, replication, autophagy, autolysosome, syntaxin-17, synaptosome-associated protein of 29 kDa, SNARE, 2BC

## Abstract

Viruses have evolved unique strategies to evade or subvert autophagy machinery. Enterovirus A71 (EV-A71) induces autophagy during infection in vitro and in vivo. In this study, we report that EV-A71 triggers autolysosome formation during infection in human rhabdomyosarcoma (RD) cells to facilitate its replication. Blocking autophagosome-lysosome fusion with chloroquine inhibited virus RNA replication, resulting in lower viral titres, viral RNA copies and viral proteins. Overexpression of the non-structural protein 2BC of EV-A71 induced autolysosome formation. Yeast 2-hybrid and co-affinity purification assays showed that 2BC physically and specifically interacted with a *N*-ethylmaleimide-sensitive factor attachment receptor (SNARE) protein, syntaxin-17 (STX17). Co-immunoprecipitation assay further showed that 2BC binds to SNARE proteins, STX17 and synaptosome associated protein 29 (SNAP29). Transient knockdown of STX17, SNAP29, and microtubule-associated protein 1 light chain 3B (LC3B), crucial proteins in the fusion between autophagosomes and lysosomes) as well as the lysosomal-associated membrane protein 1 (LAMP1) impaired production of infectious EV-A71 in RD cells. Collectively, these results demonstrate that the generation of autolysosomes triggered by the 2BC non-structural protein is important for EV-A71 replication, revealing a potential molecular pathway targeted by the virus to exploit autophagy. This study opens the possibility for the development of novel antivirals that specifically target 2BC to inhibit formation of autolysosomes during EV-A71 infection.

## 1. Introduction

Enterovirus 71 (EV-A71) is the major etiological agent of hand, foot and mouth disease (HFMD) frequently seen in young children and has also been implicated in severe neurological manifestations, including meningitis, encephalitis, and acute flaccid paralysis [1]. EV-A71 belongs to the *Picornaviridae* family, and consists of a single-stranded RNA of positive polarity. The length of its genome is approximately 7.4 kilobases. This virus encodes a single polyprotein that is proteolytically cleaved to a P1 region consisting of four structural proteins (VP1, VP2, VP3, and VP4), a P2 region consisting of non-structural proteins 2A, 2B, and 2C, and a P3 region consisting of non-structural proteins 3A, 3B, 3C, and 3D. Several precursors exist prior to cleavage, including VP0, 2BC, 3AB, and 3CD. The maturation of an immature viral particle into infectious viral particle involves the RNA-mediated cleavage of VP0 into VP2 and VP4 [2]. The genomic replication of enteroviruses occurs at cytosolic membranous vesicles. The vesicles observed during late infection resemble the membranous compartments induced by cellular autophagy [2,3,4].

The autophagy pathway is a catabolic lysosome-dependent process that is triggered by various intracellular stimuli including nutrient deprivation, misfolded protein aggregation and infections [5,6]. Autophagy begins with the formation of membranes called phagophores, which mainly depends on Beclin-1/VPS34 activity. Phagophores elongate up to the fusion of both membrane extremities, trapping cytosolic contents in newly formed double membrane-containing vesicles, known as autophagosomes [6]. The presence of microtubule-associated protein 1 light chain 3-II (LC3-II) on the autophagosome membrane is the hallmark of autophagy. During the early activation of autophagy, cytosolic LC3-I is cleaved and conjugated with the lipid phosphatidylethanolamine, converting it to the membrane-bound form, LC3-II [7,8]. Autophagosomes can fuse with late endosomes to generate amphisomes, which integrate vacuolar ATPases and contribute to intracellular vesicle acidification [9,10]. Autophagosomes or amphisomes can then fuse with lysosomes to generate autolysosomes with single membrane morphology. The cytosolic cargo sequestered within the lumina of autolysosomes, including LC3-II and long-lived proteins such as sequestosome-1 (SQSTM1/p62), are then degraded [10,11,12]. Autophagosome maturation that leads to degradation of cargo by lysosomal proteases is termed autophagic flux [13]. Autophagosome-lysosome fusion can be achieved through interaction with *N*-ethylmaleimide-sensitive factor attachment receptor (SNARE) proteins [14]. These SNARE proteins are syntaxin-17 (STX17), vesicle-associated membrane protein-8 (VAMP8) and synaptosome-associated protein of 29 kDa (SNAP29). Recently, the LC3-II-PLEKHM1-Rab7 complex was also found to mediate autophagosome-lysosome fusion [15,16]. Autophagic flux can be impeded by drugs which inhibit vesicle acidification, including the vacuolar ATPase inhibitor bafilomycin A1 (BAF-A1), as well as lysosomotropic compounds, such as chloroquine (CQ) and ammonium chloride (NH_4_Cl) [17,18,19]. The presence of regulatory mechanisms (such as the SNARE-mediated pathways) acting on late stages of autophagy implies that under certain circumstances, the generation of autophagosomes may not necessarily lead to autolysosome formation.

During late infection with poliovirus (PV), another member of the *Picornaviridae* family, double-stranded RNA (dsRNA) from the membranous viral RNA replication complex co-localizes with autophagosomes [20]. Other studies have found that autophagic vesicles are optimally produced in PV-infected cells at 5 h post-infection throughout the cytoplasm [21]. These vesicles contain both LC3-II and the lysosomal-associated membrane protein 1 (LAMP1), which indicates that the fusion of autophagosomes with lysosomes is not compromised upon PV infection [21]. In the context of EV-A71 infection, the autophagy machinery which is activated in infected cells contributes to the production of the virus in vitro and in vivo [22]. However, whether formation of autolysosomes during EV-A71 infection is required and allowed to proceed remains unknown. Furthermore, the non-structural proteins of EV-A71 that could trigger the autophagic machinery have yet to be identified.

In the present study, we show that EV-A71 triggered autolysosome formation to mediate virus replication. The STX17, SNAP29, LC3B, and LAMP1 proteins were all important for the production of infectious EV-A71. The non-structural protein 2BC was identified as the viral protein that triggers formation of autolysosomes via its interaction with STX17 and SNAP29. Taken together, our study results provide new molecular insights into EV-A71 infection, particularly the role of the late maturation stage of autophagy in viral replication.

## 2. Materials and Methods

### 2.1. Reagents (Chemicals, Antibodies, and Small Interfering RNA)

BAF-A1, NH_4_Cl, E-64d protease inhibitor, leupeptin, and pepstatin A were purchased from Merck. Rapamycin, 3-methyladenine (3-MA), and CQ were obtained from Sigma (St. Louis, MO, USA). Monoclonal antibodies targeting LC3B, LAMP1, and β-actin were purchased from Cell Signaling Technology (Danvers, MA, USA), polyclonal antibody targeting GFP fusion protein (anti-GFP) without HRP conjugation, and monoclonal antibodies targeting SQSTM1/p62 and SNAP29 were purchased from Abcam (Cambridge, UK). Monoclonal antibodies targeting STX17 were obtained from Sigma. Anti-EV-A71 monoclonal antibody that targets both VP0 and VP2 (MAB979) structural proteins was purchased from Millipore (Billerica, MA, USA). Anti-EV-A71 polyclonal antibody that targets 2C and 3A was produced by immunizing rabbits with synthetic peptides of the non-structural proteins. Control, STX17, SNAP29, LC3B, and LAMP1 siRNAs (small interfering RNA) were purchased from Santa Cruz Biotechnology (Dallas, TX, USA).

### 2.2. Cell Lines and Viruses

Human rhabdomyosarcoma (RD; ATCC no. CCL-136) and human embryonic kidney (HEK-293; ATCC no. CRL-1573 and HEK293T; ATCC no. CRL-3216) cells were maintained in complete media containing Dulbecco’s modified Eagle’s medium (DMEM) (HyClone, Logan, UT, USA), while human cervix adenocarcinoma (HeLa; ATCC no. CCL-2) cells were maintained in Eagle’s minimum essential medium (EMEM), supplemented with l-glutamine and 10% fetal bovine serum (FBS) (Gibco, Billings, MT, USA) plus penicillin/streptomycin (200 U/mL), and cultured at 37 °C in a 5% CO_2_ incubator. RD and HEK-293 cells stably expressing LC3 (HEK-293/LC3) or both LC3 and LAMP1 (RD/LC3/LAMP1) were obtained as described below. All the stable cells were grown under similar conditions as the original cell line. At 80% confluence, cells were trypsinized with 0.25% trypsin (HyClone) and subcultured in the complete medium. RD cells were used for infection experiments, and RD, HEK293 and HEK293T cells were used for transfection experiments. Cell lines were then infected with EV-A71 strains UH1/PM/1997 (GenBank accession number AM396587), 41 (a gift from Tan Eng Lee, Singapore Polytechnic, Singapore, GenBank accession number AF316321), or 41-eGFP at the indicated multiplicity of infection (MOI). Unless otherwise stated, the UH1 strain was used in all experiments. The PV type 1 vaccine strain was obtained from the Diagnostic Virology Laboratory, University Malaya Medical Centre, Kuala Lumpur, Malaysia. After an hour, the unbound viruses were removed from the cells and then cultured with fresh medium supplemented with 2% FBS. Cells were harvested at the indicated times after viral infection.

### 2.3. Cytotoxicity Analysis

The cytotoxicity of CQ, BAF-A1, and NH_4_Cl was determined using the CellTiter 96 Aqueous One solution proliferation assay reagent (Promega, Madison, WI, USA). Briefly, these compounds were added at the indicated concentrations to overnight-cultured RD or HEK-293 cells, which were then incubated for the indicated time intervals. Subsequently, 20 µL of the proliferation assay reagent was added to each well of the 96-well plate. The plate was then analyzed at the absorbance of 490 nm after 2 h of incubation at 37 °C.

### 2.4. Plaque Assay

EV-A71 and PV viral stocks were titrated in RD cells. For the collection of intracellular virus, the infected cells treated with chemicals were washed with phosphate buffered saline (PBS) and collected in 0.5 mL PBS supplemented with 100 µg/mL MgCl_2_ (Merckmillipore, Billerica, MA, USA) and 100 µg/mL CaCl_2_ (Merck). The collected cells were then lysed by one cycle of freeze-thawing. After an hour of virus adsorption, the unbound viruses were removed and cells were overlaid with 0.9% carboxymethylcellulose (Sigma) in 2% FBS DMEM. After 72 h, the overlaid medium was removed and the cells were fixed with 3.7% formaldehyde followed by staining with crystal violet.

### 2.5. Construction of Plasmids

2B, 2C, 2BC, 3A, and 3AB genes of EV-A71 were chemically synthesized from GenScript and cloned into the BamHI and EcoRI sites of pEGFP-N1 vector (Clontech, Mountain View, CA, USA) to generate GFP fusion proteins. The STX17 gene was cloned into pGBKT7 (Clontech) and pCherry (Addgene, Cambridge, MA, USA).) vectors while the 2BC gene of EV-A71 was cloned into the pACT2 (Clontech) and pDEST27 (Thermo, Waltham, MA, USA) vectors. The pmRFP-LC3 and LAMP1-YFP vectors were obtained from Addgene. The tandem tagRFP-eGFP-LC3 vector was purchased from Thermo. The EV-A71 41-eGFP infectious cDNA clone was constructed by cloning the full-length genome of the virus with eGFP into pCR-XL-TOPO (Invitrogen, Carlsbad, CA, USA) as previously described [23].

### 2.6. DNA and siRNA Plasmid Deliveries

For DNA transfection, RD, HEK-293, HEK293/LC3 and RD/LC3/LAMP1 cells were grown in 6-well plates and seeded at a density of 3 × 10^5^ cells per well 24 h prior to transfection. The cells were transfected using Lipofectamine LTX with a total of 2.5 µg of DNA plasmid unless otherwise stated. For transduction of DNA vector, RD cells were infected with 100 MOI of baculovirus bearing the construct for 72 h. For transient knockdown of gene expression, 40 nM of siRNA construct (unless otherwise stated) was transfected using Lipofectamine 2000 into the host cells for 48 h.

### 2.7. Sodium Dodecyl Sulfate Polyacrylamide Gel Electrophoresis and Western Blotting

Cells were lysed in radioimmunoprecipitation assay (RIPA) buffer containing 50 mM Tris pH 8, 150 mM NaCl_2_, 0.1% SDS, 0.5% sodium deoxycholate, 1% Triton X-100 and protease (Sigma) and halt phosphatase inhibitors cocktail (Thermo). Lysed cells were electrophoresed by either 8% or 15% sodium dodecyl sulfate polyacrylamide gel electrophoresis (SDS-PAGE) and transferred to a 0.2 μM pore size polyvinylidene fluoride membrane (Millipore). The membrane was incubated with antigen pretreatment solution (Thermo) for 10 min prior to blocking with 5% bovine serum albumin (BSA, Merck) in 0.1% Tween-20 Tris buffered saline (TBS) for 30 min. This was followed by the incubation of membrane with the indicated primary antibody diluted in primary antibody diluent (Thermo) for 1 h. The membrane was washed twice with 0.1% Tween-20 in TBS. Subsequently, the membrane was incubated with secondary antibody (HRP or IRDye conjugated) diluted in 2% BSA TBS 0.1% Tween-20. Finally, the membrane was washed twice before chemiluminescent development with Clarity Western ECL Substrate (Bio-Rad, Hercules, CA, USA) or without any substrate development. Images were captured with either BioSpectrum Imaging System (UVP, Cambridge, UK) or Odyssey SA Infrared Imaging System (Licor, Lincoln, NE, USA). The Western blot bands were quantified with Image Studio software.

### 2.8. Yeast Two-Hybrid Screening

A total of 47 autophagy-associated cDNAs were cloned into pGBKT7 from pDONR vector and transferred into the prey strain, while the 2BC gene of EV-A71 was cloned into pACT2 from pDONR vector and transferred into yeast bait strain AH109. Yeast cells were mated and subsequently plated on a selective medium lacking both leucine and histidine to determine the interaction-dependent transactivation of the *HIS3* reporter gene [24].

### 2.9. Co-Affinity Purification (Co-AP)

Each expression vector (1.5 µg) was transfected into HEK293T cells for 48 h. After cell lysis, Glutathione (GST)-sepharose 4B beads (GE Healthcare, Chalfont St Giles, UK) were used for the co-affinity purification (co-AP) [24]. GST-tagged 2BC and FLAG-tagged STX17 were detected using anti-GST and anti-FLAG monoclonal antibodies, respectively.

### 2.10. Co-Immunoprecipitation (Co-IP)

RD cells were washed twice with PBS pH 7.4 to remove traces of media. Cells were lysed with freshly prepared RIPA buffer supplemented with protease and Halt phosphatase inhibitors. Lysates were vortexed overnight at 4 °C. Following centrifugation, the clarified lysates were incubated with primary antibodies or control IgG bound Protein G Dynabeads (Invitrogen) for at least 3 h at 4 °C with end-over-end rotation. The antibody-bound magnetic beads were then washed 5 times with PBS. Binding partners of target proteins were eluted by incubating the beads with RIPA buffer containing SDS loading buffer for 10 min at 95 °C. The eluted proteins were separated on 15% SDS-PAGE and transferred on polyvinylidene fluoride (PVDF) membrane. Specific monoclonal antibodies were used to identify the binding partners of target proteins.

### 2.11. TaqMan Quantitative Real-Time PCR Assay (TaqMan qPCR)

Forward primer 5′-GAGCTCTATAGGAGATAGTGTGAGTAGGG-3′, reverse primer 5′-ATGACTGCTCACCTGCGTGTT-3′, and TaqMan probe 5′-6-carboxyfluorescein (FAM)-ACTTACCCA/ZEN/GGCCCTGCCAGCTCC-Iowa Black FQ-3′ were used to quantify EV-A71 as previously described [25]. Intracellular viral RNA from infected cells was extracted by using QIAmp viral RNA Mini Kits (Qiagen, Hilden, Germany) according to the manufacturer’s instructions. The TaqMan real-time reverse transcription (RT)-PCR assay was performed using the StepOne Plus Real Time System (ABI, Foster City, CA, USA) with the TaqMan Fast Virus 1-step master mix (ABI). The synthesis of cDNA from RNA began with reverse transcription for 5 min at 50 °C, followed by amplification for 40 cycles at 95 °C for 3 s and 60 °C for 30 s.

### 2.12. Immunofluorescence Assay

Infected and transfected cell lines were fixed with 4% paraformaldehyde prepared in PBS for 20 min at room temperature and permeabilized with 0.25% Triton X-100 (Sigma) for 10 min. The cells were then blocked with Image-iT FX signal enhancer (Invitrogen) for 1 h. Primary and subsequently secondary antibodies were incubated in the cells for 1 h, respectively. To visualize nuclei, the cells were counterstained with 4′,6-diamidino-2-phenylindole (DAPI, Sigma) for 5 min. The slides were washed 3 times with PBS and covered with coverslips containing ProLong Gold antifade reagent (Invitrogen). The images were then analyzed using a Leica TCS SP5 confocal microscope and its associated software (Leica Microsystems, Wetzlar, Germany). Co-localization was analyzed using ImageJ software with Manders coefficient plug-in. Pearson’s correlation coefficients were calculated and reported as Rr values.

### 2.13. Transmission Electron Microscopy

Infected and transfected cell lines were fixed with 2.5% glutaraldehyde in 0.1 M sodium phosphate buffer (pH 7.4) for 1 h at room temperature. The cells were harvested and fixed with fresh 2.5% glutaraldehyde for at least 4 h at 4 °C, prior to post-fixation in 2% osmium tetroxide. The cells were then dehydrated with sequential washes in 50%, 70%, 90%, 95%, and 100% ethanol and embedded in resin. The areas that consist of cells were block mounted and thinly sliced before grid staining and analyzing using a HT7700 (Hitachi, Tokyo, Japan) or 1400 EX (Jeol, Tokyo, Japan) transmission electron microscope.

### 2.14. Statistical Analysis

The data presented are the means ± standard deviations (SD) obtained from three independent experiments. Error bars were used to represent the SD. Comparisons between groups were determined by either unpaired Student’s *t*-test, two-way, or one-way ANOVA using GraphPad Prism (version 6.0, GraphPad Software, San Diego, CA, USA). A *p* value of <0.05 was considered statistically significant.

## 3. Results

### 3.1. Formation of Autolysosomes during EV-A71 Infection Is Important for Viral Replication

**Figure 1:**
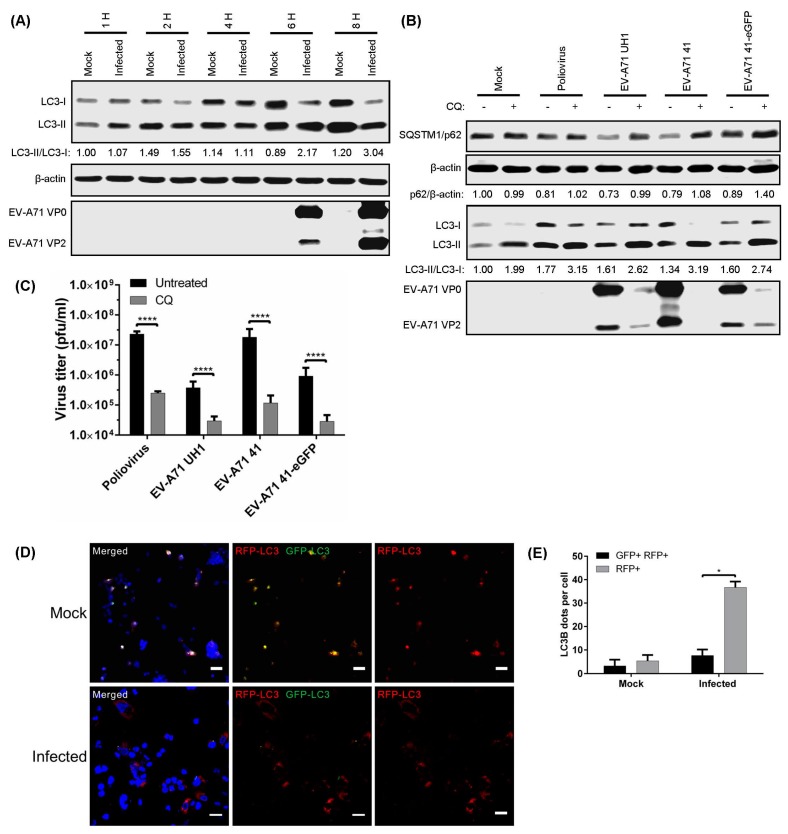
Acidic autolysosome formation triggered by enterovirus A71 (EV-A71) facilitates viral replication. Rhabdomyosarcoma (RD) cells were infected with EV-A71 (multiplicity of infection (MOI) = 10) up to various time points; then, (**A**) microtubule-associated protein 1 light chain 3 (LC3) and viral proteins were detected by Western Blot (WB). RD cells were infected with either poliovirus (PV), EV-A71 strain UH1, EV-A71 strain 41, or EV-A71 41-eGFP reporter (MOI = 10) and treated with 50 µM CQ from 1 hpi to 8 hpi; then, cell lysates were collected to determine (**B**) the sequestosome-1 (SQSTM1)/β-actin and LC3-II/LC3-I ratios by WB; and (**C**) viral titres; (**D**) RD cells were transduced with baculovirus bearing tandem RFP-GFP-LC3 for 72 h, and infected with EV-A71 for 8 hpi; then, a confocal microscope was used to detect RFP and GFP signals. Scale bar, 30 μM; (**E**) The amounts of GFP and RFP dots of LC3B per cell were determined for mock– and EV-A71-infected RD cells. Error bars, means ± SD of 3 independent experiments. One-way and two-way ANOVA or Student’s *t*-test: * *p* < 0.05, and **** *p* < 0.0001.

Among the methods used to monitor autophagy is the tracking of LC3-I to LC3-II conversion by western blot [13,26,27]. During late EV-A71 infection, a gradual increase in the LC3-II/LC3-I ratio was observed from 6 hpi to 8 hpi, and this was accompanied by an increase in the expression of viral proteins (Figure 1A).

The impairment of autolysosome formation is known to lead to the accumulation of SQSTM1/p62, a long-lived protein otherwise selectively degraded by autophagy, indicating the successful halting of protein degradation [28]. PV was used in our study as a positive control as a recent study showed that PV infection induces autolysosome formation [29]. CQ treatment increased SQSTM1/p62 protein levels at 8 hpi in PV and EV-A71-infected cells compared to non-treated infected cells (Figure 1B). The increase in SQSTM1/p62 indicates that CQ inhibited autophagic flux in virus-infected RD cells. Since LC3-II accumulation could result from either de novo formation of autophagosomes or a blockage of autolysosome formation, the LC3-II/LC3-I ratio was measured upon treatment with CQ in infected cells. As for SQSTM1/p62, CQ treatment augmented the accumulation of LC3-II at 8 hpi in PV and EV-A71-infected cells (Figure 1B). Interestingly, CQ-mediated inhibition of autophagic flux reduced the expression of EV-A71 VP0 and VP2 structural proteins. Additionally, CQ treatment also inhibited plaque formation of PV and all EV-A71 strains (UH1, 41, and 41-eGFP) by >90% (reduced by 0.99, 0.90, 0.99, and 0.95 log_10_ pfu/mL, respectively) (Figure 1C). As seen with CQ, both BAF-A1 and NH_4_Cl treatments increased the protein levels of SQSTM1/p62 and LC3-II in EV-A71-infected cells compared to non-treated infected cells (Figure S1A). Similarly, viral proteins, viral titres and viral RNA were reduced following treatments with BAF-A1 and NH_4_Cl, suggesting that vesicle acidification is important for efficient EV-A71 replication (Figure S1A–C). Lysosomal protease inhibitors including E-64d plus pepstatin A (an inhibitor of cysteine and aspartyl proteases) and leupeptin (an inhibitor of cysteine, serine, and threonine proteases) did not alter viral RNA and viral titres in EV-A71-infected cells (Figure S1D,E) [13].

To further monitor autophagosome maturation during EV-A71 infection, a tandem RFP-GFP-LC3 construct was used, which allowed for the discrimination between autophagosomes and autolysosomes. The acid-sensitive GFP is quenched in autolysosomes retaining mostly the acid-insensitive RFP, whereas both fluorochromes are visible in autophagosomes [13]. A significantly higher amount of RFP signals compared to GFP was observed in EV-A71-infected RD cells at 8 hpi compared to mock-infected cells, which suggests the formation of autolysosomes during EV-A71 infection (Figure 1D,E). Taken together, these findings indicate that EV-A71 induces autolysosome formation, which is required for viral replication.

### 3.2. Inhibition of the Autolysosome Formation Inhibits Virus RNA Replication

**Figure 2:**
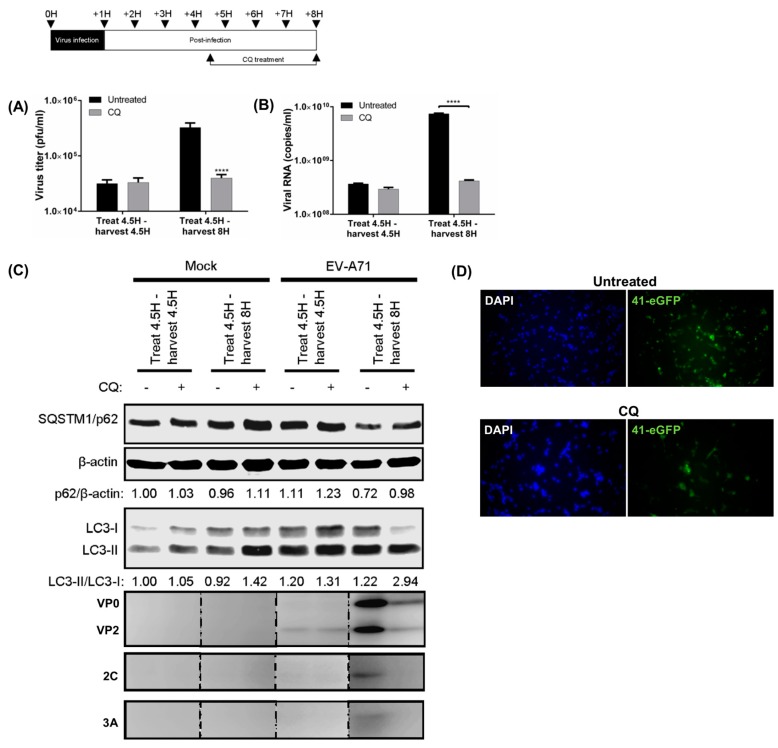
Inhibition of autolysosome formation inhibits virus RNA replication. RD cells were infected with EV-A71 (MOI = 10), and 50 μM chloroquine (CQ) was added at 4.5 hpi; then, cellular lysates were immediately harvested or collected at 8 hpi for WB to determine (**A**) viral titres; (**B**) viral RNA copies; and (**C**) the SQSTM1/β-actin, LC3-II/LC3-I ratios and viral proteins; (**D**) EV-A71 41-eGFP reporter (MOI = 10) was transfected and treated with 50 µM CQ from one hpi to 8 hpi, and the eGFP signal was observed with fluorescence microscope. Error bars, means ± SD of 3 independent experiments. One-way ANOVA and Student’s *t*-test: **** *p* < 0.0001.

We further investigated the effects of inhibition of autolysosome formation on the virus life cycle. To limit any possible effects of CQ on EV-A71 entry and initiation of virus transcription or translation, CQ was added to infected cells after 4.5 h [29]. The presence of CQ from 4.5 to 8 hpi showed similar impairment of autophagic flux as well as reduced EV-A71 viral titres and viral RNA (Figure 2A,B). The VP0 and VP2 capsids, as well as 2C and 3A proteins were greatly reduced upon treatment with CQ (Figure 2C). These data showed that the inhibitory effect of CQ on autophagosome maturation begins at 4.5 hpi, when active viral RNA replication takes place.

The complete viral genome bearing the eGFP reporter gene is often utilized to visualize virus gene expression. Using an EV-A71 41-eGFP reporter for infection, we found that a lower eGFP signal was observed in CQ treated-transfected cells, suggesting that blocking of autolysosome formation reduced the expression of the eGFP-virus (Figure 2D). Collectively, these data suggest that inhibition of autolysosome formation affects viral RNA replication and subsequent steps in the virus production.

### 3.3. 2BC Non-Structural Protein of EV-A71 Triggers Autolysosome Formation

**Figure 3:**
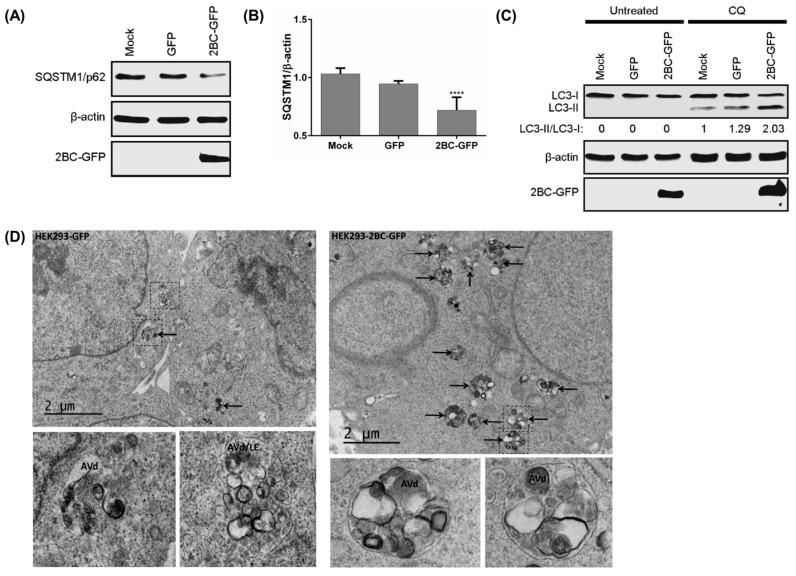
2BC non-structural protein of EV-A71 triggers autolysosome formation. (**A**) HEK-293 cells were transfected with eGFP-tagged 2BC for 48 h to detect sequestosome-1 (SQSTM1/p62) and GFP fusion proteins by Western Blot; and (**B**) SQSTM1/β-actin ratio was determined; (**C**) HEK-293 cells were transfected as in (**A**) for 24 h and treated with 50 μM chloroquine (CQ) for another 24 h prior to (**C**) WB; (**D**) HEK293/LC3 stable cells were transfected with 2BC as in (**A**) for transmission electron microscopy. AVd and black arrows indicate late autophagic vacuoles while LE indicates late endosomes. AVd/LE denotes putative amphisomes, organelles resulting from fusion of autophagic vacuoles with multivesicular late endosomes. Error bars, means ± SD of 3 independent experiments. One-way ANOVA: **** *p* < 0.0001.

To identify whether 2BC non-structural proteins of EV-A71 induce autophagic flux, 2BC was transfected into HEK-293 cells. The expression of eGFP-tagged 2BC viral protein significantly decreased the accumulation of SQSTM1/p62 compared to mock and eGFP control cells (Figure 3A,B). The presence of CQ in 2BC-transfected cells also increased the accumulation of LC3-II when compared to CQ-treated mock and eGFP control cells (Figure 3C). The expression of 2B, 2C, 3A, and 3AB non-structural proteins tagged with eGFP did not increase the accumulation of LC3-II in the presence of CQ when compared to the eGFP-tagged 2BC viral protein (Figure S2A,B). The treatment of CQ also showed no significant cytotoxicity in HEK-293 cells compared to non-treated cells (data not shown). Electron microscopy of 2BC-eGFP-HEK293/LC3 cells revealed greater accumulation of late autophagic vacuoles (AVd) 48 h after transfection compared to the control cells (Figure 3D). Taken together, these results suggest that 2BC non-structural proteins induce the formation of autolysosomes by facilitating the generation and maturation of autophagosomes.

### 3.4. 2BC Non-Structural Protein Interacts with STX17 and SNAP29, and Transient Knockdown of STX17 and SNAP29 Inhibits Production of Infectious EV-A71

**Figure 4:**
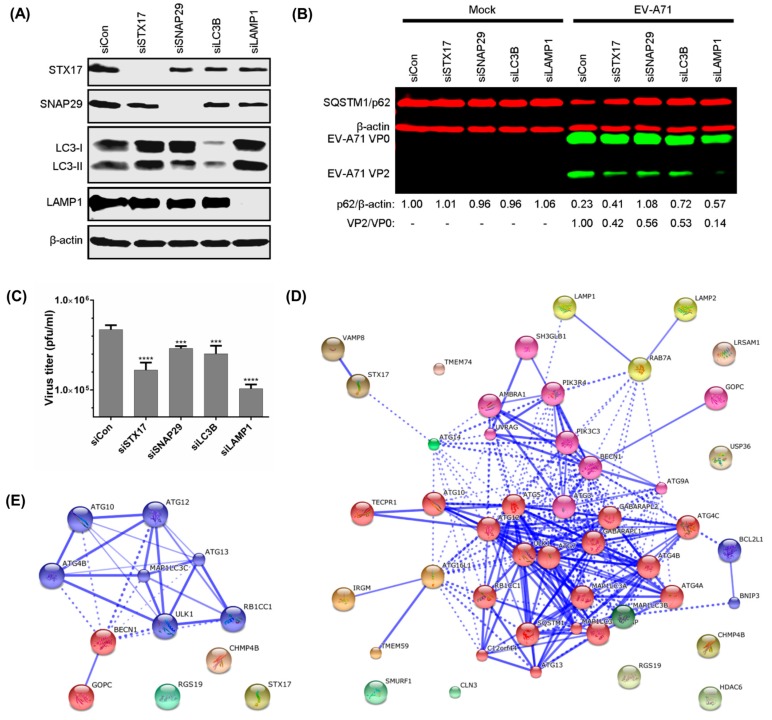
Transient knockdown of syntaxin-17 (STX17), synaptosome-associated protein of 29 kDa (SNAP29), LC3B, and lysosomal-associated membrane protein 1 (LAMP1) impairs production of infectious EV-A71. (**A**) RD cells were transfected with 40 nM of STX17, SNAP29, LC3B, LAMP1, or control pools of 3 siRNAs for 48 h, and endogenous proteins were detected by Western Blot (WB); (**B**) RD cells were transfected as in (**A**) and infected with EV-A71 (MOI = 10) for 8 hpi, and SQSTM1/p62 and viral proteins were detected by WB; (**C**) RD cells were transfected and infected as in (**B**) for plaque assay to determine viral titres; (**D**) Graphical presentation of the 47 autophagy-associated proteins considered in this study. These proteins were used in the yeast two-hybrid arrays against 2BC non-structural protein; (**E**) Graphical presentation of the 12 autophagy-associated proteins that interacted with 2BC non-structural protein of EV-A71. The Markov Cluster Algorithm was performed using STRING to cluster the autophagy-related proteins. The width of blue lines is proportional to the strength of interactions between proteins, with greater width indicating stronger interaction. The dashed lines indicate the possible interactions between proteins. One-way ANOVA: *** *p* < 0.001, and **** *p* < 0.0001.

STX17, SNAP29 [14], and LC3B [13,15,16] are proteins involved in the fusion between autophagosomes and lysosomes, while LAMP1 is a lysosomal membrane protein [30]. Transient knockdowns were performed in RD cells with pools of 3 specific siRNAs and these inhibited the expression of STX17, SNAP29, LC3B, and LAMP1 by more than 85% (Figure 4A). Silencing of these genes also increased the accumulation of SQSTM1/p62 by impairing autophagic flux during EV-A71 infection. Additionally, the production of infectious EV-A71 was also impaired following these transient knockdowns, with siLAMP1 (VP2/VP0 = 0.14) causing the highest inhibition followed by siSTX17, siLC3B, and siSNAP29 (VP2/VP0 = 0.42, 0.53, 0.56, respectively) compared to control siRNA (VP2/VP0 = 1.00) (Figure 4B). The ratio of VP2/VP0 indicates the ratio of mature/immature EV-A71 viral particles. Following treatment with specific siRNAs, the production of EV-A71 infectious progeny was measured by plaque assay. Compared to the control siRNA, transient knockdown of STX17, SNAP29, LC3B, and LAMP1 significantly impaired EV-A71 plaque formation (reduced by 0.45, 0.21, 0.27, and 0.66 log_10_ pfu/mL, respectively) (Figure 4C). Thus, these findings further indicate that the production of infectious EV-A71 requires components of the autophagosome-lysosome machinery.

**Figure 5:**
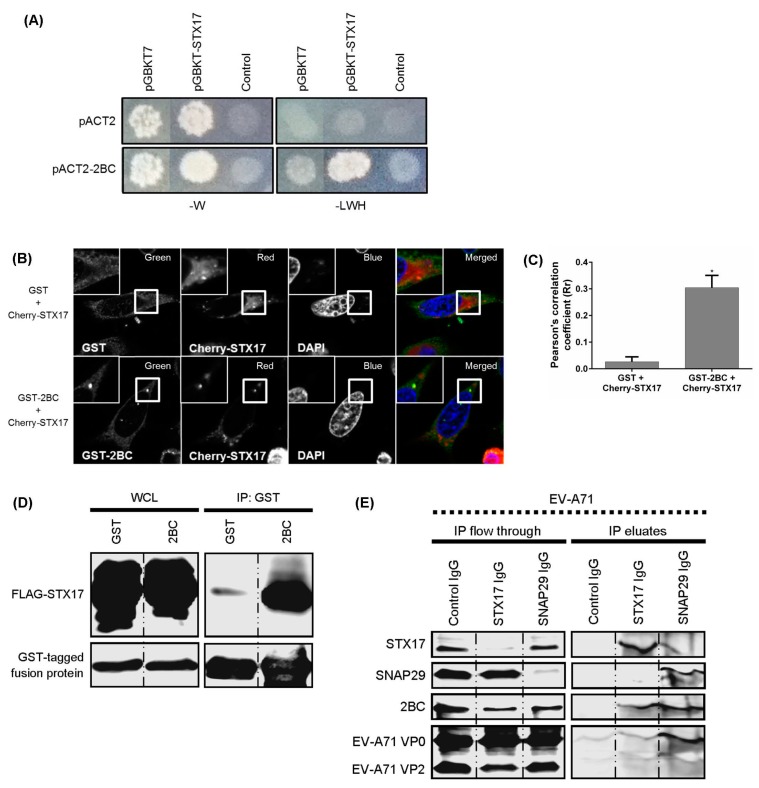
The 2BC non-structural protein of EV-A71 interacts with syntaxin-17 (STX17) and synaptosome-associated protein of 29 kDa (SNAP29). (**A**) Yeast two-hybrid assays of 2BC and STX17. Yeasts were transformed with pGBKT7 or pGBKT7-STX17 together with pACT2 or pACT2–2BC. Yeasts were then grown on selective medium without tryptophan (-W) or without leucine, tryptophan, and histidine (-LWH); (**B**) HeLa cells were transfected with pDEST27 or pDEST27–2BC in combination with pCherry-STX17 for 48 h. The transfected cells were examined under a confocal microscope with objective 63× (scale bar 10µm) for the presence of co-localization; and (**C**) the Pearson’s correlation coefficient was determined; (**D**) HEK-293T cells were co-transfected with FLAG-STX17 and GST-2BC for 48 h. Lysates were collected for co-immunoprecipitation (IP) using glutathione (GST) sepharose. Western Blot was performed to detect FLAG-STX17 and GST fusion proteins; (**E**) RD cells were infected with EV-A71 for 8 hpi and lysates of infected cells were then harvested prior to co-immunoprecipitation (co-IP) assay using anti-STX17 and anti-SNAP29. The final IP eluates and discarded IP flow through were collected for WB to detect 2BC (anti-2C polyclonal antibody) and structural proteins (mAB979) of EV-A71. One-way ANOVA: * *p* < 0.05.

To determine the underlying mechanism by which the 2BC non-structural protein of EV-A71 facilitates the formation of autolysosomes, 2BC was used as a bait protein in a yeast two-hybrid system against an autophagic matrix containing 47 different human proteins involved in the autophagy process (Figure 4D,E) [24]. The 2BC non-structural protein was found to interact with STX17 and 11 other autophagy-related proteins (ATG10, ATG4B, ATG12, ATG13, MAP1LC3C, BECN1, ULK1, RB1CC1, GOPC, RGS19, and CHMP4B; Figure 4E). In view of the involvement of STX17 in autolysosome formation and its function in the production of infectious EV-A71, STX17 was selected for further investigation. STX17 was found to physically and specifically interact and co-localize with 2BC in transfected HEK-239T and HeLa cells (Figure 5A–D). To further investigate the interaction of 2BC or viral structural proteins with STX17 and SNAP29 during EV-A71 infection, co-immunoprecipitation was performed using lysates of infected cells. In addition to STX17, 2BC interacted with SNAP29 (final IP eluates) compared to control immunoglobulin (Ig)G (Figure 5E). The IP flow through further showed the reduction of STX17 and SNAP29 in infected cell lysates after co-immunoprecipitation, indicating the successful pull-down of these proteins. Interestingly, VP0, the immature structural protein of EV-A71, was also identified as a new binding partner of SNAP29. Taken together, these results indicate that 2BC non-structural protein of EV-A71 interacts with both STX17 and SNAP29.

## 4. Discussion

We have shown here that EV-A71 triggers autolysosome formation in RD cells. Inhibition of autophagosome-lysosome fusion impaired the replication and production of EV-A71 at the step of virus RNA replication. The induction of autophagic flux is also not strain-specific. Parallel to our findings, hepatitis C virus (HCV) requires the autophagy machinery to facilitate viral translation and replication [31]. Infection of host cells with coxsackievirus B3 requires only the early stage of autophagosome formation, and not the late maturation stage of autophagosomes, for productive replication [32]. For PV infection, autophagosome maturation does not affect translation, but inhibits virus RNA replication and promotes maturation of viral particles [29]. In both poliovirus and EV-A71 infections, the inhibition of autophagosome maturation reduced the viral titres, indicating that this process is important in the life cycles of these closely related enteroviruses. A recent study showed that Beclin-1, class III PI3K Vps34, *N*-glycanase (NGLY1) and the valosin-containing protein (VCP), which function in autophagosome formation, also facilitate the replication of EV-A71 [33]. Therefore, EV-A71 could utilize multiple steps in the autophagic machinery to benefit its replication.

The maturation of autophagosomes requires v-ATPases, and these decrease the pH of the vesicles. The acidic environment in the vesicles is crucial for the activation of hydrolases and proteases, which are involved in the degradation of incoming cargo [13]. Several viruses have developed unique mechanisms to thrive in these acidic compartments. PV triggers autophagosome maturation and this process mediates the cleavage maturation of VP0 into VP4 and VP2 structural proteins. However, inhibition of lysosomal proteases did not facilitate the production of PV infectious progeny [29]. We found that three inhibitors of vesicle acidification (CQ, BAF-A1, and NH_4_Cl) inhibited the expression level of viral proteins, viral RNA replication, and viral titres albeit at different sensitivities. Similar to PV, treatment with inhibitors of lysosomal proteases did not influence the production of infectious EV-A71.

The 2BC viral protein of PV triggers LC3 conversion, and in combination with 3A viral protein induces autolysosome accumulation [21,26]. The 2C non-structural protein of coxsackievirus A16 (CV-A16) triggers the formation of autophagosomes [34]. In our study, we found that the 2BC non-structural protein of EV-A71 is capable of inducing autolysosome accumulation in transfected cells. However, the specific functional domains of 2BC that facilitate the accumulation of autolysosomes are yet to be identified. In support of our findings, a recent study has shown that the expression of neither 2B, 2C, 3A, 3B, 3AB, 3C nor 3D individually can trigger autophagy, revealing the potential unknown role of other precursor proteins in this machinery [35]. Although we observed that 2BC plays a role in the accumulation of autolysosomes, other viral proteins may synergistically contribute to this process during EV-A71 infection.

The recent identification of STX17 and SNAP29 as SNAREs involved in the formation of autolysosomes led us to study their involvement in EV-A71 infection. STX17 localizes to the autophagosome membrane while SNAP29 is the adaptor protein that links STX17 to lysosomal SNARE [14,15,16]. Additionally, the homotypic fusion and protein sorting (HOPS) is a tethering factor that has an identical function to SNAP29, mediating fusion between STX17 and the lysosomal transmembrane protein [36]. A recent study has found that HCV inhibits autolysosome formation by reducing the expression of STX17, which increases the production of infectious HCV viral particles [37]. In contrast, we found that silencing of STX17, SNAP29, LC3B, and LAMP1 inhibited autophagic flux and reduced the production of infectious EV-A71. The presence of low virus titres rather than complete inhibition may be attributed to an alternative mechanism of autophagosome-lysosome fusion via the LC3-PLEKHM1-RAB7 machinery [15,16]. We further found that 2BC non-structural protein of EV-A71 specifically interacts with STX17 and SNAP29, and this reaffirms the importance of STX17 and SNAP29 in EV-A71 infection. Yeast-2 hybrid and co-affinity purification assays further show that 2BC interacts with STX17. The findings from co-immunoprecipitation assays further indicate that 2BC binds to STX17 as well as SNAP29 in RD cells to regulate autolysosome formation. Importantly, the immature structural protein of EV-A71, VP0 also interacts with SNAP29. This interaction between VP0 and SNAP29 opens the possibility for further investigation into its role in EV-A71 infection.

As autophagy also regulates innate immune responses [38], the interplay between EV-A71, autophagy and innate immunity should be further explored. Many autophagy proteins have now been shown to have non-autophagic roles. Both ATG13 and FIP200, components of the ULK complex that regulate upstream of the autophagy pathway via mTOR, have no beneficial effect on EV-A71 replication in U20S cells, suggesting that some autophagy-related proteins have unconventional functions in specific cell lines [39].

In conclusion, we have shown in this study that EV-A71 infection triggers autolysosome accumulation in vitro. The formation of autolysosomes enhances the expression of viral proteins, viral replication, and production of infectious EV-A71 during the virus RNA replication step. This study opens the possibility for the development of novel antivirals that specifically target 2BC to inhibit formation of autolysosomes during EV-A71 infection.

## Supplementary Materials

The following are available online at www.mdpi.com/1999-4915/9/7/169/s1, Figure S1: Virus yields are reduced with inhibitors of vesicle acidification but not lysosomal protease inhibitors. RD cells were infected with EV-A71 (multiplicity of infection, MOI = 10) and treated with either 50 μM chloroquine, 50 nM bafilomycin A1, or 20 mM ammonium chloride from 1 hpi to 8 hpi, after which sequestosome-1 (SQSTM1)/p62 and viral proteins were detected by Western Blot. RD cells were infected and treated as in (**A**); then, (**B**) TaqMan qPCR was performed to detect viral RNA and (**C**) plaque assay performed to detect viral titres. RD cells were infected with EV-A71 (MOI = 10) and treated with 20 μM leupeptin or 10 μg/mL E-64d + 10 μg/mL pepstatin A. After 8 hpi, cells were harvested for (**D**) TaqMan qPCR and (**E**) plaque assay. Error bars, means ± Standard deviation of 3 independent experiments. One-way and two-way ANOVA or Student’s *t*-test: * *p* < 0.05, ** *p* < 0.01, and **** *p* < 0.0001, Figure S2: EV-A71 non-structural proteins 2B, 2C, 3A, and 3AB are not involved in autophagosome maturation. HEK-293 cells were transfected with GFP-tagged 2BC and (**A**) 2B and 2C or (**B**) 3A and 3AB DNA-expressing plasmids. Cells were then treated with 50 μM chloroquine or untreated and incubated at 37 °C for 24 h. Cell lysates were harvested for immunoblotting to detect LC3-II and GFP-tagged viral proteins by using rabbit anti-LC3B and mouse anti-GFP-HRP monoclonal antibodies, respectively.
