# Supplementary figures and images for: 2BC Non-Structural Protein of Enterovirus A71 Interacts with SNARE Proteins to Trigger Autolysosome Formation
Source: Viruses. 2017 Jul 4;9(7):169. doi: 10.3390/v9070169 (PMC5537661; doi:10.3390/v9070169)

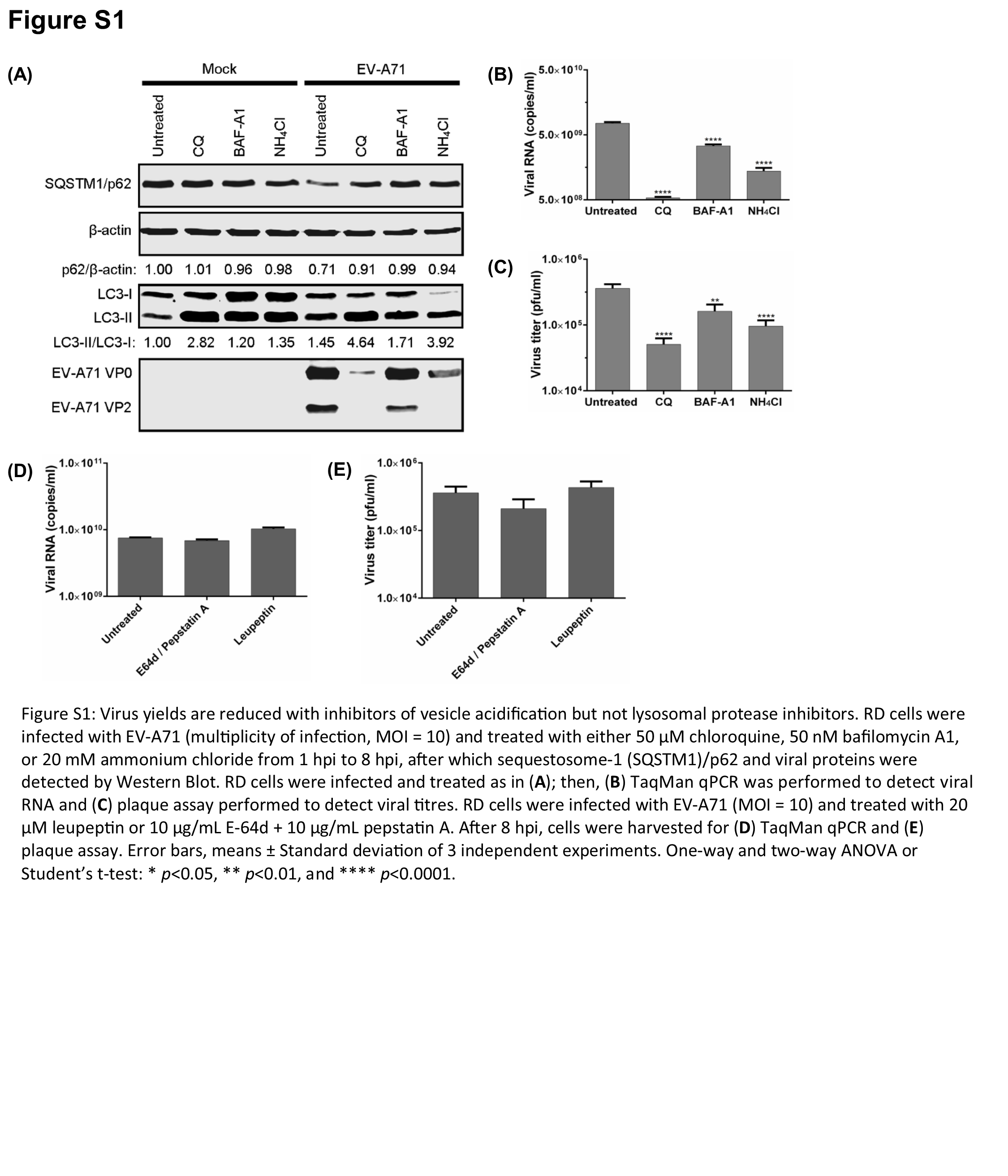

Supplement: Supplementary file 1 [file viruses-09-00169-s001.zip › Fig S1 proofread.tif]

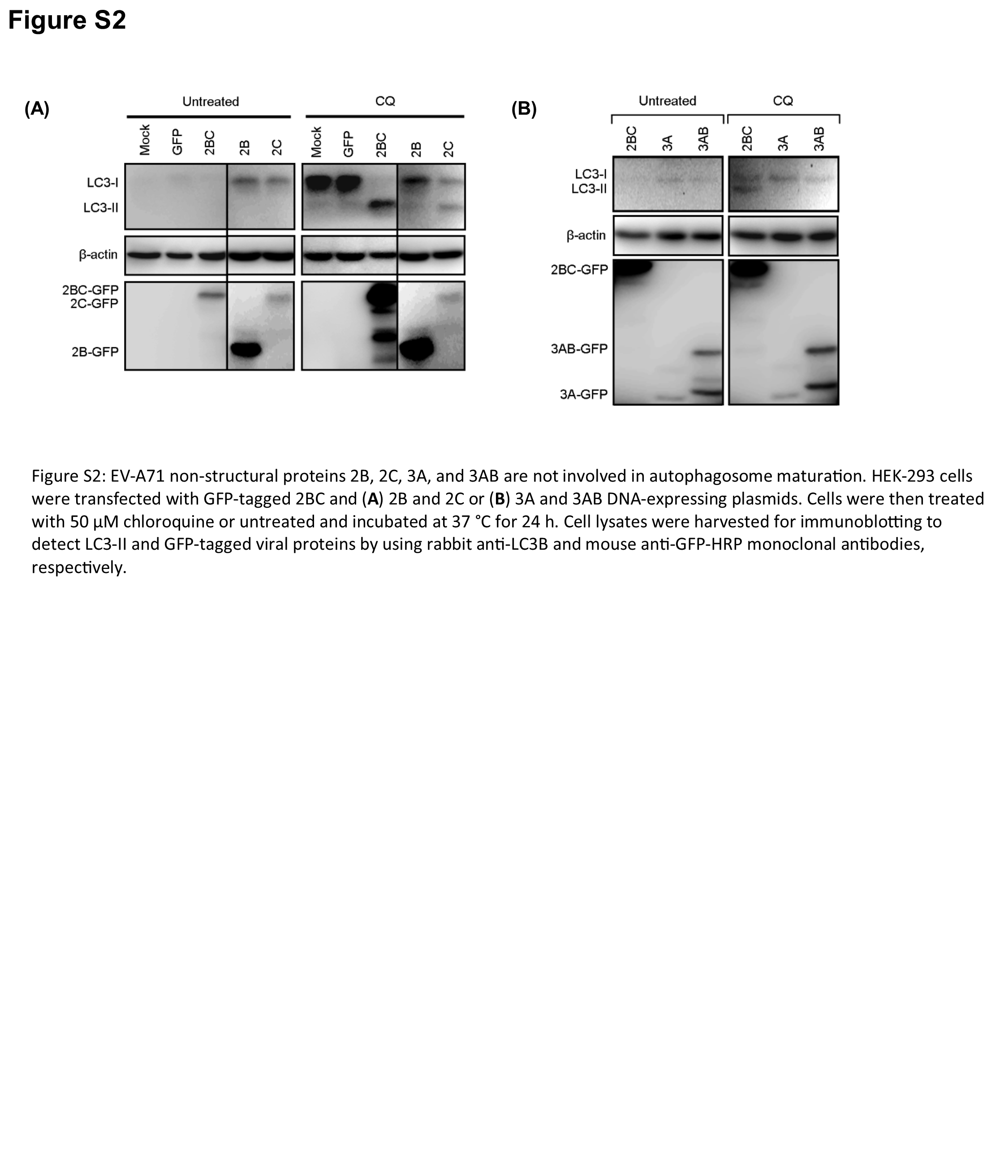

Supplement: Supplementary file 1 [file viruses-09-00169-s001.zip › Fig S2 proofread.tif]
